# Resting State-fMRI with ReHo Analysis as a Non-Invasive Modality for the Prognosis of Cirrhotic Patients with Overt Hepatic Encephalopathy

**DOI:** 10.1371/journal.pone.0126834

**Published:** 2015-05-14

**Authors:** Wei-Che Lin, Tun-Wei Hsu, Chao-Long Chen, Cheng-Hsien Lu, Hsiu-Ling Chen, Yu-Fan Cheng

**Affiliations:** 1 Department of Diagnostic Radiology, Kaohsiung Chang Gung Memorial Hospital and Chang Gung University College of Medicine, Kaohsiung, Taiwan; 2 Department of Biomedical Imaging and Radiological Sciences, National Yang-Ming University, Taipei, Taiwan; 3 Department of Radiology, Taipei Veterans General Hospital, Taipei, Taiwan; 4 Department of Surgery, Kaohsiung Chang Gung Memorial Hospital and Chang Gung University College of Medicine, Kaohsiung, Taiwan; 5 Department of Neurology, Kaohsiung Chang Gung Memorial Hospital and Chang Gung University College of Medicine, Kaohsiung, Taiwan; Affiliated Hospital of North Sichuan Medical College, CHINA

## Abstract

**Background:**

To investigate the relationships among regional activity abnormalities, clinical disease severity, and prognosis in cirrhotic patients with overt hepatic encephalopathy (OHE) using resting-state functional magnetic resonance imaging (rs-fMRI).

**Methods:**

Regional homogeneity (ReHo) values of 12 cirrhotic patients with OHE and 12 age- and sex-matched healthy volunteers were calculated from rs-fMRI. Two-sample t-test was performed on individual ReHo maps between the two groups. The relationships between ReHo variation, disease severity, and prognosis were analyzed.

**Results:**

Cirrhotic patients with OHE had significantly low ReHo values in the left middle cingulum, bilateral superior temporal, left inferior orbito-frontal, right calcarine, left inferior frontal gyrus, left post-central, left inferior temporal, and left lingual areas, and high ReHo in the right superior frontal, right inferior temporal, right caudate, and cerebellum. There was significant group difference in the right superior temporal lobe (p=0.016) and crus1 of the left cerebellum (p=0.015) between survivors and non-survivors in the OHE group. Worse Glasgow Coma Scale was associated with increased local connectivity in the left cerebellar crus I (r= -0.868, p=0.001).

**Conclusions:**

Information on the functional activity of cirrhotic patients with OHE suggests the use of rs-fMRI with ReHo analysis as a non-invasive prognosticating modality.

## Introduction

Overt hepatic encephalopathy (OHE) is a common complication of chronic liver disease, occurring in 30–45% of patients with cirrhosis [1]. Aside from being difficult to manage, it is also associated with increased mortality. There have been extensive investigations on the pathophysiology of OHE with the aim of developing effective therapies to prevent its onset. But because it is not yet well understood and difficult to predict, the failure rate of therapy remains high.

Cerebral edema leading to increased intracranial pressure (ICP) is a major complication of patients with OHE [2]. It is characterized by the presence of astrocyte swelling [3]. By quantification of brain metabolites and grade of edema, magnetic resonance imaging (MRI) has been proposed to be an *in vivo* marker of prognosis in fulminant hepatic failure [4]. The results from some studies also suggest that patients with OHE may have disturbed global or regional brain energy metabolism and intracranial hemodynamics [5, 6]. Increased cerebral ammonia uptake, impaired metabolism, and decreased glucose utilization occur in several brain regions in patients with OHE, with significant alterations depending on the severity of hepatic encephalopathy [7]. These data suggest that abnormalities in cerebral metabolism and hemodynamics may be a contributing factor to HE and may serve as predictors for the evolution of HE.

Recently, functional neuroimaging studies have reported a decline in functional connectivity within the default mode network (DMN) [8] and the thalamocortical network [9] in unconsciousness subjects. The DMN has also been extensively evaluated and is thought to represent the neural consciousness stream [10–12] and to be associated with different degrees of impaired cognition and consciousness [13–15]. Patients with OHE may have a mild lack of awareness and be lethargic and somnolent, and they may be arousable or in a coma, and these symptoms and even death have also been demonstrated to be associated with reduced functional connectivity in the DMN [10].

Furthermore, recent neuro-imaging studies in cirrhotic patients have described early impairment of the neural connectivity mechanism and abnormal coupling between different cortical areas, regardless of whether or not the patients had OHE [11, 12]. Disruptions in inter-regional brain connectivity have been reported to lead to failure of functional integration within the brain, and this may partly account for the deficits in cognition and behavior in patients with cirrhosis [13]. However, specific associations between loss of consciousness and outcomes of patients with OHE and impaired inter-regional synchronization remain unclear.

Recently, resting-state functional connectivity has been measured using task independent functional MRI to examine changes in synchronized low frequency oscillations in blood-oxygen-level dependence (BOLD) signal during resting states. These can identify functionally interconnected brain regions [14]. Previous studies have demonstrated that inter-cortical functional connectivity and small-world topology may be altered proportional to the severity of OHE and degree of brain edema [15].

Regional homogeneity (ReHo) is a newly developed method for evaluating the similarities or coherence of intra-regional spontaneous low-frequency (<0.08 Hz) BOLD signal fluctuations in voxel-wise analysis across the entire brain [16]. It is now thought that regional synchrony of BOLD activity can explain the variance in neurovascular coupling and task activations [17]. This method has also been successfully used to investigate functional modulations in the resting state of patients without OHE to detect the progression of cognitive changes in cirrhotic patients [11, 12]. However, little is known about alterations in regional synchrony of BOLD activity in patients with OHE and their relationship to disease outcome.

Based on neuropathology and neuroimaging, we hypothesized that patients with OHE may experience modulation of neural activity with alterations in brain ReHo. To test this hypothesis, we first used rs-fMRI to explore differences in ReHo between patients with cirrhosis and an acute episode of OHE and normal controls. Second, we examined possible relationships between areas with significant differences in ReHo in the two groups and clinical variables. The goal of this study was to determine whether ReHo can help to predict outcomes in patients with OHE.

## Materials and Methods

### Participants

From August 2009 to December 2010, fifteen patients with cirrhosis and an acute episode of overt hepatic encephalopathy were recruited and those who were clinically stable underwent imaging protocol. Patients who had unsatisfactory image acquisition (motion artifacts due to encephalopathy) were excluded. Twelve patients (eight with hepatitis B and four with hepatitis C; seven males and five females; mean age, 56.67 ± 8.57 years; age range, 40–69 years) who fulfilled these criteria were included.

Patients with liver cirrhosis were diagnosed according to the reduced Child-Pugh score [18] and imaging features [19]. Overt HE (OHE) was graded by the West Haven criteria [20]. All patients underwent laboratory screening, including albumin, creatinine, bilirubin, prothrombin time, international normalized ratio, aspartate aminotransferase, and serum venous ammonia levels, on the same day as the MRI scan.

For comparison, 12 healthy volunteers (seven men and five women; mean age, 50.33 ± 11.28 years; age range, 32–70 years), without any medical history of neurologic disease, were recruited within the hospital and served as the control group. Kaohsiung Chang Gung Memorial Hospital and Chang Gung University College of Medicine, Kaohsiung institutional Ethics Committee approved the study. Because the study re-analyzed MRI data from a previous research in which written consent was already given by the patients for their information to be stored and used for research, the ethics committee waived the need for informed consent. All data were analyzed anonymously.

### MR data acquisition and pre-processing

#### Data acquisition

Functional imaging data were acquired using a 3.0 T GE Signa MRI scanner (Milwaukee, WI, USA). Resting-state images taken from 300 contiguous echo planar imaging whole brain functional scans (TR = 2 s, TE = 30 ms, FOV = 240 mm, flip angle 80°, matrix size 64 x 64, thickness 4 mm) were collected.

During the resting experiment, the normal controls were instructed to relax with their eyes closed, but without falling asleep. After the examination, they were asked questions to verify the degree of their cooperation as previously reported by Lv et al [11]. We also asked the patients with OHE to follow these instructions during scanning. Head movements were minimized using customized cushions. Monitoring of vital parameters including electrocardiography, blood pressure, pulse oxymetry and respiratory rate in the OHE patients was performed by a radiologist throughout the experiment. A 3D high-resolution T_1_-weighted anatomic image was also acquired using an inversion recovery fast spoiled gradient-recalled echo pulse sequence (TR = 9.5 ms; TE = 3.9 ms; TI = 450 ms; flip angle 20°; field of view 256 mm; matrix size 512 x 512).

#### Resting-state fMRI pre-processing and individual analyses

Prior to pre-processing, the first 10 volumes were discarded to reach a steady-state magnetization and allow the participants to adapt to the scanning noise [11, 15, 21]. Resting-state fMRI data pre-processing was then performed using the Statistical Parametric Mapping (SPM8, Wellcome Department of Cognitive Neurology, London, UK; http://www.fil.ion.ucl.ac.uk/spm/) and Data Processing Assistant for rs-fMRI (DPARSF) tools [22]. To control motion-induced artifacts, point-to-point head motion was estimated for each subject [23, 24]. Three patients were excluded because of head motion more than 2.0 mm or 2° cumulative translation or rotation. We also used frame-wise displacement and temporal derivative of the fMRI time series reported by Power et al [23] to calculated the rigid body realignment derivatives that are used to realign BOLD data during fMRI preprocessing. In our study we removed volumes with head mean absolute displacements >0.5 mm and BOLD signal displacements >0.5% prior to analysis of regional homogeneity calculation results. The standard Montreal Neurological Institute template provided by SPM was further used for normalization with re-sampling to 2 mm cubic voxels and a Gaussian kernel of 6 mm (full width at half maximum) for spatial smoothing. The waveform of each voxel was finally used to remove the linear trend of time course and for temporal band-pass filtering (0.01 to 0.08 Hz) to reduce low-frequency drift and high-frequency physiological high-frequency respiratory and cardiac noise [25, 26].

### Statistical analysis

#### Data processing and regional homogeneity calculation

The ReHo was used to analyze rs-fMRI data. The indexes of locally synchronous activity was measured by calculating the voxel-wise similarity of activity fluctuations within a given voxel time-course. The ReHo values indicated regionally localized temporal synchronization within the cluster at different spatial scales and depended directly on the neighbors’ cluster size. The computation of ReHo at rest was as previously described [16]. Briefly, Kendall’s coefficient of concordance (KCC) for each voxel in the brain was calculated voxel-wise by applying a cluster size of 26 voxels according to the following formula:

$$W=\frac{\sum {\left(R_{i}\right)}^{2}-n{\left(\bar{R}\right)}^{2}}{\frac{1}{12}K^{2}(n^{3}-n)}$$

where W was the KCC of given voxels, ranging from 0 to 1, *Ri* was the rank sum of the *ith* time-point;$\bar{R}$ = ((n + 1)K)/2 was the mean of the *Ri*, K was the number of time-series within a measured cluster (n = 27; one given voxel plus the others inside the cluster), and n was the number of ranks (corresponding to time-points; 300 minus 10 = 290 ranks after discarding the 10 volumes). From this equation, an individual ReHo map was obtained. For standardization purposes, each individual ReHo map was divided by that subject’s global mean brain KCC value to minimize inter-individual variability for statistical analysis.

To highlight the characteristic regional homogeneity spatial pattern of each participant group, a one-sided one-sample t-test (*p* < 0.05, false discovery rate correction [FDR]) was performed. A random-effect two-sample t-test was then performed on the individual ReHo maps between the OHE and control groups. A customized explicit mask was obtained from the patients’ and controls’ un-modulated grey matter tissue maps. The results are displayed at *p* < 0.05 using AlphaSim correction (with a combination of a threshold of *p* < 0.01 and a minimum cluster size of 40 voxels). This correction was conducted using the AlphaSim function of REST software (http://www.restfmri.net), which applied Monte Carlo simulation to calculate the probability of false positive detection by taking both the individual voxel probability threshold and cluster size into consideration [22]. To exclude the effects of confounding covariates on ReHo values, age, sex, and total brain volume were included as covariates in the analysis. Covariates of age, sex, and brain volume were regressed out using the DPARSF software toolbox [22]. We also calculated Cohen’s d values for the mean values within each cluster, because effect size emphasizes the size of the difference rather than confounding this with the sample size.

Mean ReHo indices representing each significant cluster were calculated for each participant and compared between survivors and non-survivors using the Mann-Whitney U test. Data was adjusted for age and sex. The relationships between ReHo indices and laboratory test results were investigated using the partial Pearson correlation analysis. After Bonferroni correction for the number of ROIs, the significance threshold for the two-tailed partial correlation tests was set at *p*<0.05 (with multiple comparisons). All statistical analyses were performed using the SPSS statistical package (V13; SPSS Inc., Chicago, IL, USA).

## Results

### Participants

The demographic and clinical characteristics of the participants are presented in Table 1. The median time to first imaging after the onset of encephalopathy was 3 days (range, 1–8 days). At the time of imaging, the mean Glasgow Coma Scale of the patients with OHE was 2.58 ± 1.16. Sedatives, muscle paralyzing agents and prophylactic anti-epileptics were not used. There were no significant differences in demographic variables and brain volume between the patients with OHE and the controls. In the OHE group, eight patients survived with conservative therapy, and the remaining four succumbed to their illness. There were significantly higher bilirubin levels *(p* = 0.001), International Normalized Ratio values *(p* = 0.043) and Score of West Haven criteria *(p* = 0.006) in the patients with OHE who died than in the patients with OHE who survived.

**Table 1:** Analysis of demographic variables and cognitive assessments between the healthy controls and patients with OHE.

Variable	Control group (n = 12)	OHE group (n = 12)	p value	OHE survivor (n = 8)	OHE non-Survivor (n = 4)	p value
Age (years)	50.33 ± 11.28	56.67 ± 8.57	0.188	58.62 ± 7.82	52.75 ± 9.81	0.284
Sex (male/female)	7/5	7/5	1.000	5/3	2/2	1.000
Education (years)	10.54 ± 3.98	9.79 ± 4.64	0.267	9.43 ± 3.71	9.92 ± 4.81	0.334
GMV (cm^3^)	583.66 ± 42.34	547.50 ± 69.60	0.328	536.00 ± 81.28	570.47 ± 35.93	0.562
WMV (cm^3^))	582.27 ± 48.10	512.10 ± 66.73	**0.010** *	524.98 ± 74.70	486.35 ± 44.52	0.612
CSFV (cm^3^))	216.61 ± 25.91	225.47 ± 41.69	0.986	2236.74 ± 37.17	202.90 ± 46.17	0.444
TIV (cm^3^)	1382.54 ± 90.92	1285.06 ± 138.42	0.067	1297.73 ± 160.42	1259.73 ± 94.49	0.946
Creatinine (mg/dL)	-	1.14 ± 1.30	-	0.833 ± 0.47	1.74 ± 2.22	0.274
AST (IU/L)	-	109.42 ± 104.96	-	66.25 ± 22.30	195.75 ± 155.98	0.195
Bilirubin (mg/dL)	-	14.80 ± 15.22	-	6.36 ± 6.79	31.68 ± 13.14	**0.001** *
Venous ammonia (mg/dL)	-	170.42 ± 97.95	-	150.00 ± 104.81	211.25 ± 78.81	0.330
Albumin (mg/dL)	-	2.84 ± 0.31	-	2.73 ± 0.29	3.05 ± 0.29	0.107
International Normalized Ratio (INR)	-	1.97 ± 0.75	-	1.67 ± 0.68	2.57 ± 0.51	**0.043** *
West Haven criteria		2.58 ± 1.16		2.00 ± 0.93	3.75 ± 0.50	**0.006** *

Means and standard deviations of raw scores for the healthy control group and the patients with OHE. For each variable, the p value indicates the significance level of the appropriate statistical test comparing the raw scores of the control group and the patients with OHE. Abbreviations: CSFV, cerebrospinal fluid volume; GMV, gray matter volume; TIV, total intracranial volume; WMV, white matter volume.

* P value < 0.05

### Resting-state cerebral connectivity changes

#### Difference in regional homogeneity between OHE and healthy controls

**Fig 1:**
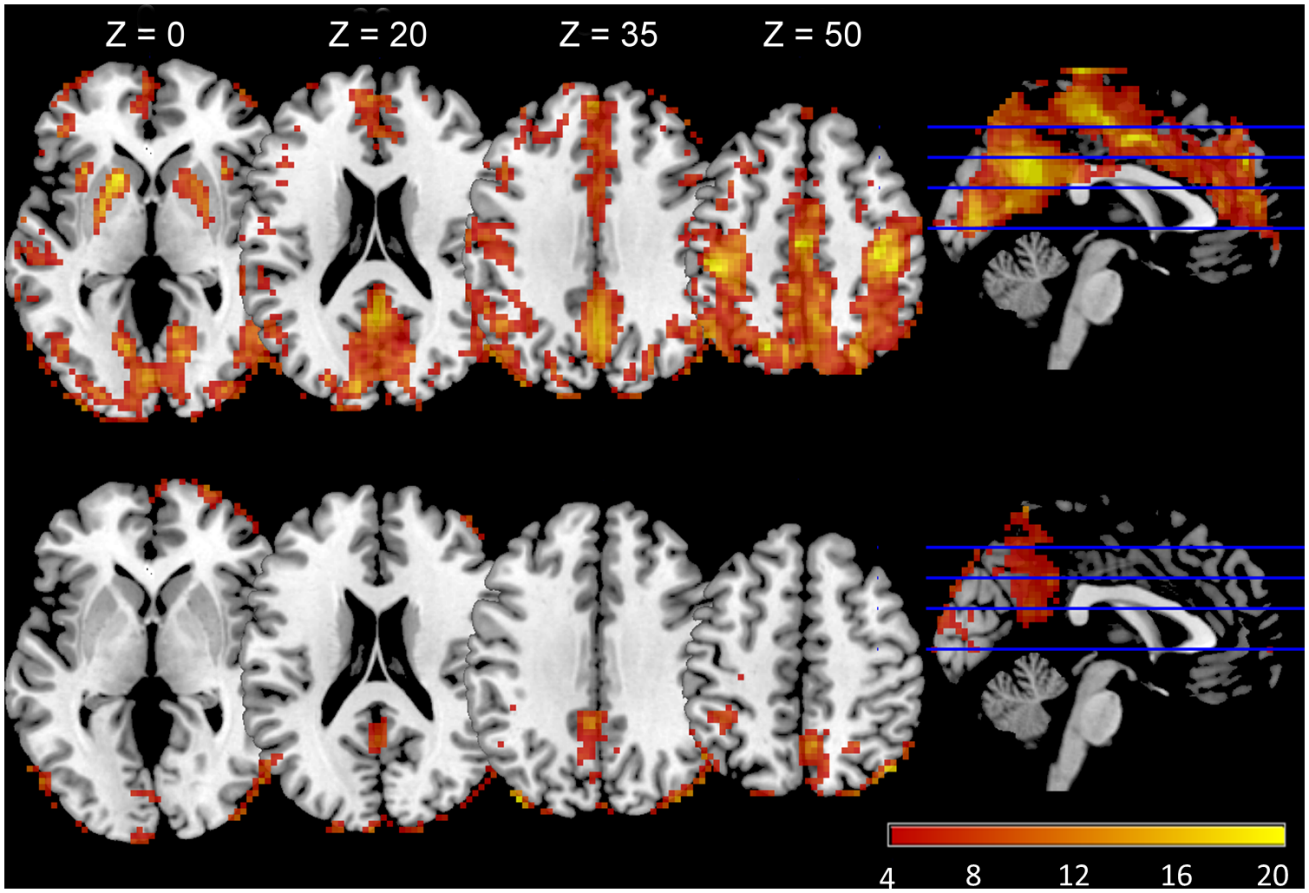
Mean ReHo maps of patients with overt hepatic encephalopathy (lower column) and healthy controls (upper column). The prefrontal cortex, lingual gyrus, middle temporal gyrus, bilateral precentral gyrus, paracentral lobule and precuneus exhibited significantly higher ReHo values than the global mean ReHo values of both groups (*p*<0.01, false discovery rate corrected), but had different strengths between the two groups.

**Fig 2:**
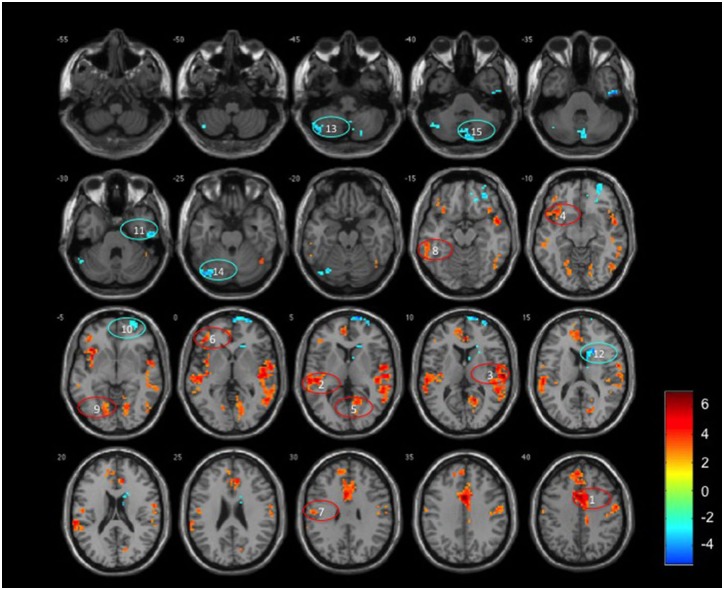
A random effect two sample t-statistical difference map between patients with overt hepatic encephalopathy (OHE) and healthy controls. The OHE patients showed significantly decreased ReHo in the (1) left middle cingulum, (2) left superior temporal gyrus, (3) right superior temporal gyrus, (4) left inferior orbito-frontal gyrus, (5) right calcarine, (6) triangular part of left inferior frontal gyrus, (7) left post-central gyrus, (8) left inferior temporal gyrus, and (9) left lingual, and increased ReHo in the (10) right superior frontal gyrus, (11) right inferior temporal gyrus, (12) right caudate, and (13) left crus II, (14) left crus I, and (15) right crus II of the cerebellum. T-score bars were shown on the right. Hot and cold colors indicated increased and decreased ReHo in OHE patients compared to controls, respectively. These criteria met a corrected threshold of *p*<0.05.

The ReHo analysis reflected the local connectivity. In both groups, we found that several brain regions had higher ReHo values than the whole-brain average, including the prefrontal cortex, lingual gyrus, middle temporal gyrus, bilateral precentral gyrus, paracentral lobule and the precuneus (Fig 1), however there were strong reductions in some components in the OHE patients. Whole brain analysis indicated that OHE subjects displayed significantly decreased ReHo values in many areas, including the left middle cingulum, bilateral superior temporal, left inferior orbito-frontal and right calcarine. There were increased ReHo regions in the right superior frontal, right inferior temporal, right caudate, and cerebellum (left crus I, II, and right crus II). The detailed results were shown in Fig 2 and Table 2 (*p*<0.05, Monte Carlo correction).

**Table 2:** Brain regions with abnormal regional homogeneity in cirrhotic patients with overt hepatic encephalopathy.

ReHo	Cluster anatomical locations	Cluster size	Peak coordinate (mm)			T value	Cohen’s d
			x	y	z		
Controls > Patients	Middle Cingulum, left	1452	-3	9	42	7.9105	5.415
	Superior Temporal, left	319	-45	-24	3	7.6436	2.815
	Superior Temporal, right	1079	60	-18	9	7.4988	4.420
	Inferior Orbito-frontal, left	126	-36	21	-6	5.6305	3.566
	Calcarine, right	183	18	-57	9	5.3771	1.904
	Inferior Frontal, triangular part, left	54	-45	36	0	4.5238	1.911
	Post-central, left	60	-54	-15	30	3.8768	1.744
	Inferior Temporal, left	67	-57	-48	-18	3.7976	1.796
	Lingual, left	53	-18	-78	-6	3.7681	1.408
Patients > Controls	Superior Frontal, right	173	18	72	6	-5.2082	2.617
	Inferior Temporal, right	45	54	-15	-33	-4.9109	2.120
	Caudate, right	68	21	18	15	-4.5425	2.228
	Cerebellum, Crus II, left	49	-45	-72	-45	-4.2793	1.831
	Cerebellum, Crus I, left	44	-42	-78	-24	-4.0780	2.209
	Cerebellum, Crus II, right	71	6	-81	-42	-3.8325	2.530

Results were according to the automated anatomical labeling software package and digital atlas of the human brain. Multiple comparisons were corrected using Monte Carlo simulations with a corrected threshold of *p*<0.05. The threshold of the cluster size was 40 calculated by the following parameters: single voxel p = 0.01, 1,000 simulations, FWHW = 6 mm, cluster connection radius = 5 mm with grey matter mask.

#### Difference in regional homogeneity between survivors and non-survivors

**Fig 3:**
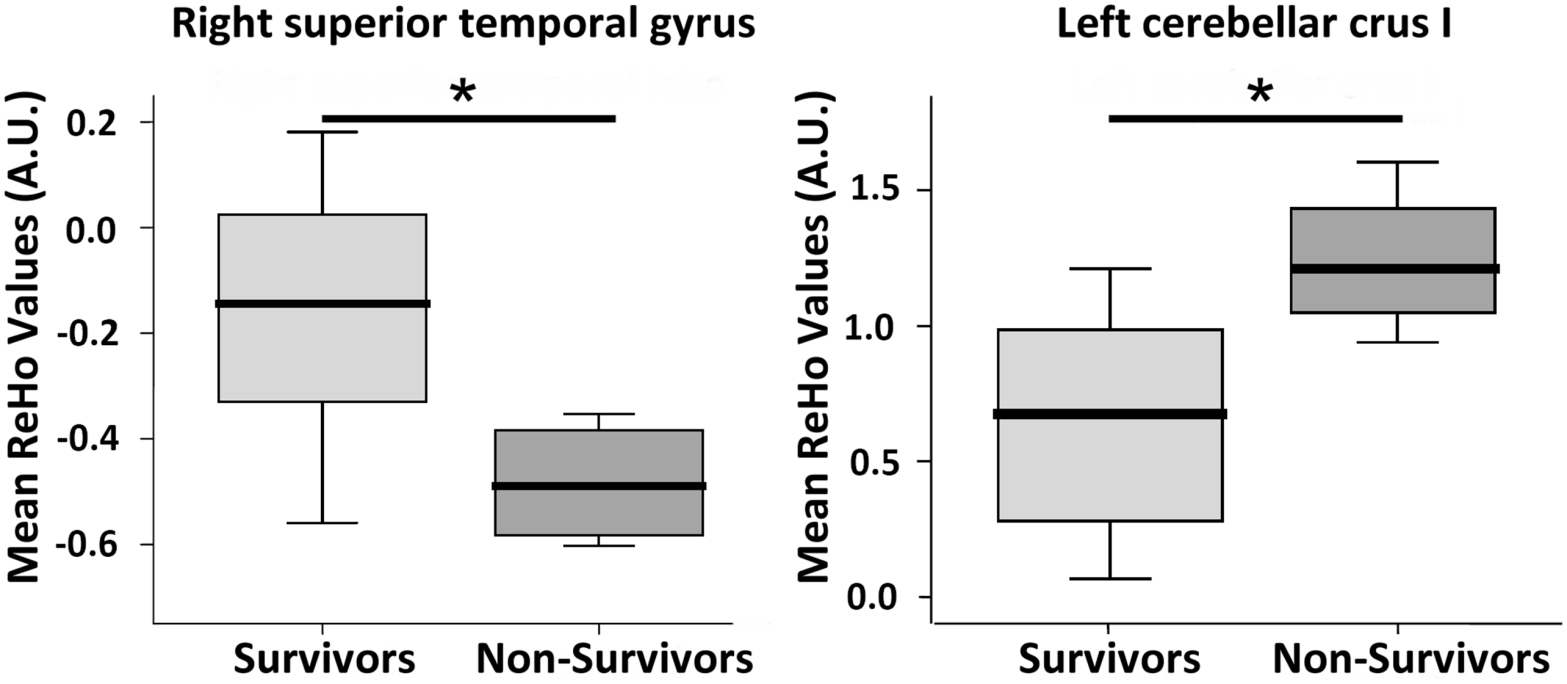
Scattergram and box plot of ReHo among eight survivor and four non-survivor cirrhotic patients with overt hepatic encephalopathy (OHE). The mean ReHo values of the right superior temporal gyrus was lower in the non-survivors compared to the survivors (-0.48 vs. -0.16, *p* = 0.016). The mean ReHo values of the left cerebellar crus I was higher in the non-survivors than in the survivors (1.24 vs. 0.66, *p* = 0.015). Boxes represent the 25th and 75th percentiles, and the lines in the boxes indicate the medians. Upper and lower lines of whiskers represent minimum and maximum values. ReHo, regional homogeneity; AU, arbitrary unit; *p<0.05

Further analysis of ReHo values derived from group-wise comparison was conducted between the two patient subgroups. Results showed significant group difference in the right superior temporal lobe (p = 0.016) and crus 1 of the left cerebellum (*p* = 0.015) (Fig 3).

#### Correlations between clinical and functional data

**Fig 4:**
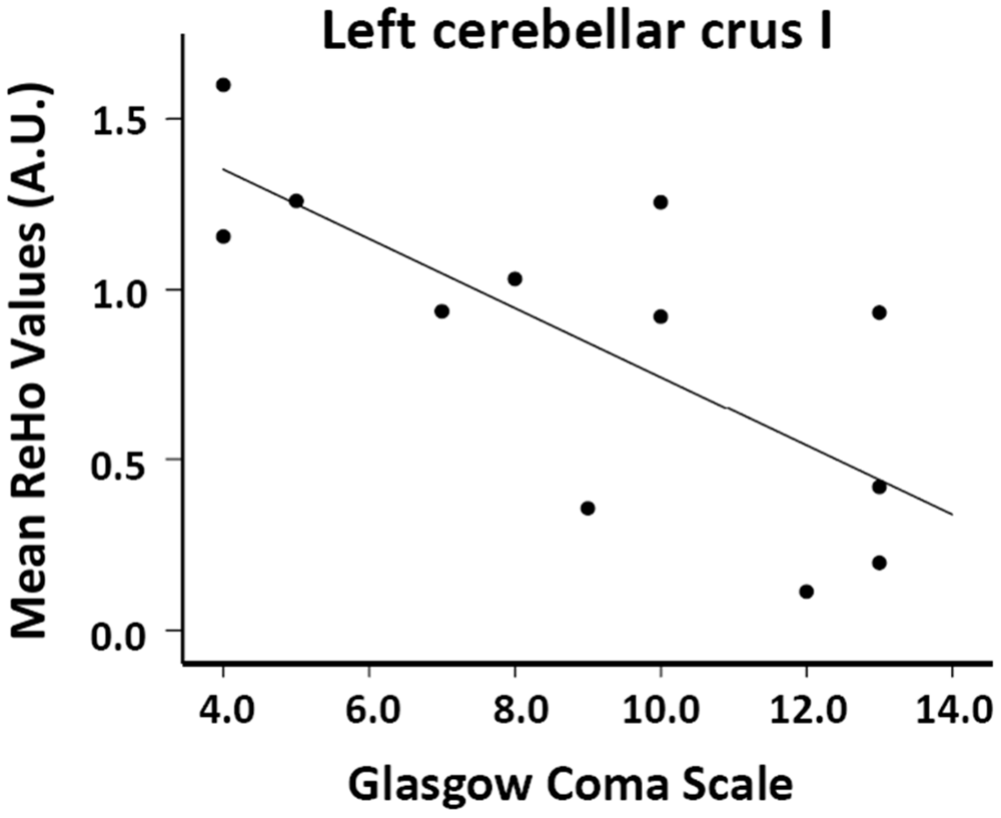
The correlation between the ReHo values and Glasgow Coma Scale in patients with overt hepatic encephalopathy. The Glasgow Coma Scale negatively correlated with ReHo values in the left cerebellar crus I.

The correlations between regional ReHo values and clinical disease profiles are summarized in Table 3. In cirrhotic patients with acute OHE, an increased creatinine value was correlated negatively with decreased local connectivity in the right calcarine (r = -0.639, *p* = 0.046) and left lingual (r = -0.658, *p* = 0.038) areas, respectively. Increase aspartate aminotransferase value correlated positively with increased local connectivity in the left cerebellar crus II (r = 0.677, *p* = 0.031), while increased bilirubin correlated positively with increased local connectivity in the left cerebellum crus I (r = 0.780, *p* = 0.008). Worse Glasgow Coma Scale was significantly positively associated with increased local connectivity in the left cerebellar crus I (r = -0.868, *p* = 0.001, multiple comparison correction) (Fig 4).

**Table 3:** Correlation between regional Reho values and clinical disease profiles.

		Creatinine (mg/dL)	AST (IU/L)	Bilirubin (mg/dL)	Ammonia (mg/dL)	Albumin (mg/dL)	INR	GCS
**Controls > Patients with OHE**								
Middle Cingulum, left	r	-0.061	0.184	0.554	-0.101	-0.035	0.479	-0.362
	p	0.868	0.612	0.097	0.780	0.924	0.161	0.303
Superior Temporal, left	r	-0.072	-0.344	-0.058	0.021	-0.584	0.171	0.025
	p	0.842	0.331	0.873	0.954	0.076	0.636	0.945
Superior Temporal, right	r	-0.264	-0.293	-0.646	-0.184	-0.425	-0.422	0.563
	p	0.462	0.411	0.044	0.610	0.221	0.225	0.090
Inferior Orbito-frontal, left	r	0.632	0.532	0.390	0.013	0.542	0.550	-0.505
	p	0.050	0.114	0.266	0.972	0.105	0.100	0.137
Calcarine, right	r	**-0.639**	-0.124	-0.615	-0.388	-0.521	-0.557	0.532
	p	**0.046**	0.733	0.059	0.268	0.122	0.094	0.113
Inferior Frontal, triangular part, left	r	0.337	0.568	0.267	0.241	0.220	0.243	-0.458
	p	0.341	0.087	0.456	0.502	0.541	0.499	0.183
Post-central, left	r	-0.047	-0.313	-0.288	0.148	-0.316	-0.130	0.209
	p	0.898	0.379	0.420	0.683	0.374	0.719	0.562
Inferior Temporal, left	r	-0.143	-0.362	0.032	-0.362	-0.573	0.367	0.198
	p	0.693	0.304	0.931	0.304	0.083	0.297	0.584
Lingual, left	r	**-0.658**	-0.102	-0.328	-0.329	-0.445	-0.398	0.328
	p	**0.038**	0.779	0.355	0.353	0.197	0.255	0.354
**Patients with OHE > Controls**								
Superior Frontal, right	r	0.100	-0.230	0.555	0.309	0.309	0.327	-0.214
	p	0.783	0.523	0.096	0.384	0.385	0.356	0.553
Inferior Temporal, right	r	0.322	0.093	0.094	0.204	-0.198	0.273	-0.316
	p	0.365	0.799	0.797	0.572	0.583	0.445	0.374
Caudate, right	r	-0.046	0.052	0.165	0.423	0.065	-0.201	-0.207
	p	0.899	0.887	0.649	0.223	0.859	0.578	0.566
Cerebellum, Crus II, left	r	0.354	**0.677**	0.117	0.082	0.438	0.060	-0.437
	p	0.316	**0.031**	0.747	0.822	0.205	0.869	0.207
Cerebellum, Crus I, left	r	0.047	0.293	**0.780**	0.525	-0.144	0.520	**-0.868**
	p	0.897	0.412	**0.008**	0.119	0.691	0.124	**0.001**
Cerebellum, Crus II, right	r	0.549	0.526	0.011	0.206	0.605	0.034	-0.315
	p	0.100	0.119	0.976	0.569	0.064	0.926	0.376

Statistical threshold was set at p<0.05 (Boldface*). Abbreviations: AST, aspartate aminotransferase; INR, International Normalized Ratio; GCS, Glasgow Coma Scale

## Discussions

The present study shows that the ReHo method can detect patterns of homogeneity changes in OHE in the resting state. There are diffused decreased ReHo values in the cortical regions and increased ReHo in the bilateral caudate and cerebellum in patients with OHE. Patients who survived and those who succumbed to their illness also exhibit differences in ReHo values in the right superior temporal lobe and crus I of the left cerebellum. Furthermore, correlation analysis indicates that the mean ReHo in most brain regions is related to disease severity on laboratory examination and the consciousness of cirrhotic patients. These findings have not been reported previously in patients with cirrhosis and an acute episode of OHE.

Functional MRI (fMRI), based on blood oxygenation level dependent (BOLD) contrast, has been used to measure neuronal activity indirectly [27]. Tasks can increase regional neuronal activity accompanied by an enhanced regional cerebral metabolic rate of oxygen, which in turn causes regional cerebral blood flow and blood volume elevation [27]. The ReHo method was developed to characterize the local synchronization of spontaneous fMRI BOLD signals in the resting state of a given voxel to the nearest neighboring voxels (typically 26 voxels) using Kendell’s coefficient of concordance (KCC) [16]. This reflects the coherence of spontaneous neuronal activity, and its contribution to task activation has been demonstrated to be due to the amplitude of low frequency fluctuations, which in part influences neuronal activity during a task and thus task activation [17, 28]. However, it was difficult to clarify the etiology of ReHo alterations in the patients with OHE in the present study. In addition to reflecting synchronization of local neuronal activity, holding one’s breath [29], regional vascular responses [30] and regional vascular network [31] have all been demonstrated to affect changes in BOLD signal. In the current study, the aberrant values of ReHo in the patients with OHE suggest that neural function/vascularity in specific brain regions was synchronized to a greater or lesser extent relative to the normal controls. Further studies are warranted to investigate the abnormalities observed in this study, although they improve the understanding of neural substrates underlying cognitive impairment in cirrhotic patients.

In the present study, significant decreases in ReHo values in the patients with OHE were observed in the middle cingulum and inferior temporal lobes involving part of the DMN (Fig 2). The functional connectivity of the DMN has been negatively associated with attention during the performance of demanding externally cued tasks. In addition, completing the DMN also correlates well with the exhibition of consciousness [32], and decreased DMN had been demonstrated to associate with different degrees of impaired cognition and consciousness [33–35]. Our findings extend these functional connectivity results by showing decreased intraregional synchronization in cortical midline regions in patients with OHE. Furthermore, our findings suggest disrupted neural synchronization during the development of OHE, which is consistent with a study on anesthesia that showed a decrease in ReHo and temporal variance of spontaneous activity in cortical midline regions [36]. Taken together, these results strongly suggest impaired inter-regional synchronization and consequently decreased communication between regions and networks in patients with OHE, and a specific association with loss of consciousness.

The present study also demonstrates extensively altered ReHo values in the association, primary, and limbic/para-limbic regions, including the primary motor and language and visual networks which is consistent with a previous study that showed decreased ReHo values in the left lingual gyrus, right middle temporal gyrus, bilateral precentral gyrus, paracentral lobule and precuneus in patients without OHE [11]. Decreased ReHo values in the temporal, calcarine and lingual gyrus have been reported to contribute to different visually associated functional deficits [37]. Our results support previous studies in that cirrhotic patients may exhibit impairment in processing visual information and top-down modulation of visuospatial selective attention in event-related potential tests [38], reduced bilateral cerebral glucose utilization in visual association areas in positron emission tomography (PET) studies [7], and altered neural interaction between regions processing visual information in task fMRI studies [39]. The clinical significant is supported by the positive association between an increased BUN value and decreased local connectivity in the right calcarine (r = -0.639, *p* = 0.046) and left lingual (r = -0.658, *p* = 0.038) areas. Furthermore, a decreased post-central gyrus ReHo value may also contribute to motor deficits in cirrhotic patients. Increasing evidence suggests that a deterioration in synchronization of neuronal activity in the motor-associated system plays an important role in motor deficits in patients with cirrhosis [40]. In our previous study, small world properties, suggesting functional network organization, were also disrupted and depended on the severity of OHE and increased impairment of liver function [15]. The origin of ReHo and the neurophysiological basis remain unclear. Nevertheless, our results implicate that inter-regional dis-synchronization and network dis-communication may underlie the clinical manifestation of deficits in OHE.

Similar to previous studies, we also found increased regional ReHo values. Compared with the normal controls, the OHE patients had increase ReHo values in the caudate and cerebellum. Chen et al observed higher synchronization of cerebellar neuronal activity in patients with minimal HE compared with normal controls [41]. The cerebellum, coordinating with the cerebrum, influences multiple functions via several cerebro-cerebellar circuits including connecting with the frontal lobe for executive function, basal ganglia for cognition and motor functions, temporal lobe for emotion and memory, and occipital lobe for visual processing [42, 43]. Therefore, an increase in cerebellar ReHo values may result from a compensatory effect due to the decrease in regional synchrony in these neocortical regions and the associated functional deficits in cirrhotic patients. Our results are also consistent with a previous study which found increased ReHo values in the caudate nucleus of patients with MHE compared to normal controls [12]. In healthy controls, Zang et al found that the pattern of ReHo values was very similar to that observed in PET [16]. Although the exact biological mechanism of ReHo remains unclear, a similar compensatory mechanism has also been reported in many other previous PET studies corresponding to blood flow and redistribution of metabolism from various cortical regions to the cerebellum and subcortical grey matter regions, including the caudate region [44, 45]. Further studies investigating ReHo by rs-fMRI and metabolic alterations by PET in patients with OHE are recommended.

An increased cerebellar ReHo value was particularly correlated with increased levels of aspartate aminotransferase (r = 0.677, *p* = 0.031) and bilirubin (r = 0.780, *p* = 0.008), and worse Glasgow Coma Scale (r = -0.868, *p* = 0.001). In addition, we also found significantly higher ReHo values in the cerebellum of patients with OHE who did not survive compared to those who survived. Cirrhotic patients with and without HE are associated with different degrees of cerebral edema which parallel disease severity and serum ammonia level [19]. By using rs-fMRI, increase brain edema has been associated with a decline in functional network in HE [13], and it is believed to be influenced by dysmetabolism of glutamate-glutamine [46]. It is also known that cytotoxic edema is caused by cerebral energy depletion as a consequence of hypoxia [47]. In patients who suffer a global cerebral hypoxic/ischaemic injury, the cerebellum can develop reversal of the normal density relationship between grey and white matter on computed tomography scans [48], suggesting reservation/improvement of cerebellum perfusion. A compensatory increase in cerebellar flow under hypoxia indicates preferential perfusion of brain regions vital to respiratory and cardiovascular stability [49]. Adequate treatment for cirrhotic patients may reverse brain edema and re-establish impaired functional connectivity with improvement in cognitive function [21, 50]. Investigations between neuronal activity and irreversible adverse outcome in patients with HE are limited. Although, the correlation between changes in ReHo values and ammonia level in this small sample size study is limited, the association between ReHo and worse disease severity on laboratory profiles and conscious status partially supports our hypothesis that an increase in synchronization of cerebellar local neuronal activity may be a consequence of dysmetabolism and altered blood flow. Evaluation of local synchronization of spontaneous fMRI BOLD signals may therefore help to predict unfavorable outcomes in patient with OHE.

There are several limitations of the current study. Our results are limited to a small sample size which may affect the statistical analysis and comprehensive interpretation of the findings. Further studies with more patients with homogeneous etiology are needed. As a cross-sectional study, we can only observe the progression from survivor to non-survivor in different subjects but not in the same patient group enrolled in a longitudinal study. Further study is warranted to verify the finding of this study. Another consideration is the cardiac and respiratory fluctuation effects resulting from slow sampling rates (repetition time of 2000 msec) which might be aliased into the low-frequency blood oxygenation level—dependent MR signal fluctuations. Elimination of this physiological noise is difficult and simultaneous cardiac recording may provide a more direct correction in future study.

## Conclusions

The ReHo method has been assessed in terms of evaluating altered resting-state properties and for providing evidence of functional abnormalities in cirrhotic patients with an acute episode of OHE. The results reveal that altered functional connectivity is located mainly in the default model network and in the association, primary, and limbic/para-limbic regions. The findings here also suggest that an abnormal ReHo index, especially in cerebellum, identified by using the rs-fMRI may be a potential biomarker of prognosis in those critical patients.
